# Involving clinicians in management: assessing views of doctors and nurses on hybrid professionalism in clinical directorates

**DOI:** 10.1186/s12913-021-06352-0

**Published:** 2021-04-15

**Authors:** Anna Prenestini, Marco Sartirana, Federico Lega

**Affiliations:** 1 Department of Economics, Management and Quantitative Methods (DEMM) and Center of Research and Advanced Education in Health Administration (CRC HEAD), Università degli Studi di Milano, Milan, Italy; 2 Centre for Research on Healthcare and Social Management (CeRGAS) and SDA Bocconi Government, Health and Not for Profit division, Bocconi University, Milan, Italy; 3 Department of Biomedical Sciences for Health (SCIBIS) and Center of Research and Advanced Education in Health Administration (CRC HEAD), Università degli Studi di Milano, Milan, Italy; 4 Center for Applied Research in Health Economics, Organization and Management, IRCCS Galeazzi, Milan, Italy

**Keywords:** Hybrid professionalism, Clinical directorate, Management, Clinician engagement, Hospital, Nurses, Doctors, Italy, COVID-19

## Abstract

**Background:**

Hybrid professionalism is one of the most effective ways to involve clinicians in management practices and responsibilities. With this study we investigated the perceptions of doctors and nurses on hybridization in clinical directorates (CDs) in hospitals.

**Methods:**

We investigated the attitudes of healthcare professionals (doctors and nurses) towards eight hospital CDs in the Local Health Authority (LHA) of Bologna (Emilia Romagna, Italy) 6 years after their implementation. We used a validated questionnaire by Braithwaite and Westbrook (2004). Drawing on Palmer et al. (2007), we added a section about the characteristics of department heads. In all, 123 healthcare professionals in managerial roles completed and returned the questionnaire. The return rate was 47.4% for doctors and 31.6% for nurses.

**Results:**

Doctors reported an increase in clinical governance, interdisciplinarity collaboration, and standardization of clinical work. Hybridization of practices was noted to have taken place. While doctors did not see these changes as a threat to professional values, they felt that hospital managers had taken greater control. There was a large overlap of attitudes between doctors and nurses: inter-professional integration in CDs fostered alignment of values and aims. The polarity index was higher for responses from the doctors than from the nurses.

**Conclusion:**

The study findings have implications for policy makers and managers: mission and strategic mandate of CDs; governance of CDs, leadership issues; opportunities for engaging healthcare professionals; changes in managerial involvement during the COVID-19 pandemic. We also discuss the limitations of the present study and future areas for research into hybrid structures.

## Background

The introduction of managerial reforms in healthcare challenged traditional professional values of autonomy and patient centricity, as well as practices of peer-based appraisal and self-regulation in healthcare professional organizations [1]. To overcome resistance from clinicians, forms of “hybrid” professionalism, i.e., managerial roles and responsibilities assigned to professionals, have been envisioned as a means to bridge the gap between the two worlds [2–4]. The involvement of doctors in management can be viewed as a potential response to societal and patient needs in a move to improve patient centeredness and efficiency, quality, and efficacy of services [5, 6]. It can foster greater collaboration among specialties, sharing of resources, and creation of healthcare pathways; it can enhance clinical governance, minimizing the friction between two apparently contrasting sets of values and cultures [7]. Yet after 30 years of experimentation, many healthcare organizations still struggle with implementing hybrid professionalism [2, 8]. Understanding the reasons behind (in) effective implementation is relevant for both theory and practice. To this end, we explored how hospital doctors and nurses view hybrid professionalism within clinical directorates, which is a paradigmatic example of how organizations can foster the engagement of clinicians in managerial practices. We wanted to determine whether attitudes to managerial involvement differed by professional role.

According to the literature on hybrid professionalism, some professionals are willing to embrace management, while others are reluctant to accept clinical governance and its systems and may even employ tactics to openly or subtly oppose it [9–11]. However, hybrid (also termed managed) professionalism has the potential for achieving meaningful synthesis of the competing values embedded in the logics of management and professionalism: the former more focused on population groups and services efficiently delivered within allocated resources, the latter more focused on individual patients, preservation of clinical autonomy, and primacy of the medical specialty [12]. This is consistent with the scholarship on how competing institutional logics can coexist and hybridize [13, 14]. Professionals were found able to blend or overlap managerial and clinical values and practices [15], bringing organization into the professional sphere [16]. A reshaped model of professional work that can interact with organizational demands is arising and gaining legitimacy among practitioners [17].

Among the various forms of hybrid professionalism described by Noordegraaf [16], the most studied is the *mixed professional*, i.e., individual professionals who work in between competing logics. What is also important to understand is how hybrid professionalism unfolds within *mixed structures*, i.e., systems that embed professionalism within organizational contexts in which organizational structures become more dominant as they restrain professional autonomies and hybridize professional work. A contextualized understanding of hybridization can be gained by studying how organizational interventions solve the conflict between professional and organizational logics and its impact on individual identities. To do this, we need to bridge the literature on hybridity and professional identities, with its typical focus on the individual (e.g., [7, 18]), with the literature on organizational change and health services, and processes of change within professional organizations (e.g., [19]).

A key aspect is the institutional and organizational arrangements through which hybridization takes place. For instance, McGivern et al. [10] found that the hybridization of professionals heading clinical directorates (CDs) differs considerably from the hybridization of professionals in managed clinical networks. At the meso/micro level, Reay and colleagues [20] reported that when business manager roles support general practitioners, they can influence the hybridization of doctors. More recently, Sartirana et al. [21] found that the development of a new identity of doctors-in-management is influenced by organizational structures and systems that favor interactions with nurses, support staff, mentors, and senior hospital executives.

With this study, we wanted to expand the current literature by analyzing the views and attitudes of doctors and nurses towards CDs, one of the main structures of hybrid professionalism in secondary and tertiary health care. Different from previous studies on rank-and-file professionals, our study population was doctors who were already in leadership positions. From this original perspective we were able to address issues specific to the Italian context, in which unit chiefs are responsible for the management of physical assets (beds, operating rooms, technologies, etc.), finance and human resources (conventionally, also nursing staff are subordinated to the heads of specialty units). Here we compared the doctors’ perspective to that of nurse managers, whose views on the managerialization of physicians has been marginally studied in the literature on hybrids. Our research questions were “*How do doctors view the development of hybrid professionalism within clinical directorates?*” and “*Do the attitudes of doctors and nurses differ?*”. Finally, while most studies used qualitative methodologies, for our study we used a mixed approach to measure diverse dimensions of hybrid professionalism via a self-report questionnaire [22] that investigates the views and attitudes of professionals towards CDs. We added a section with open-ended questions to the questionnaire.

### Clinical directorates

Various different types of hybrids have been implemented in organizational structures. CDs in hospitals have been found to be one of the finest examples of how doctors can take managerial positions in hospital organizations [23, 24]. These intermediate management structures group clinical specialties together to improve clinical governance, efficiency [25], resource management, decision-making [26, 27] and service delivery [28]. CDs can foster a style of interaction and interdisciplinary teamwork that is more collaborative than the conventional hierarchy of following “doctor’s orders” [29] because it reduces the disciplinary self-referentiality that creates “tribes” in the clinical organization [30]. They are headed by a clinical director, i.e., a medical professional who coordinates the activity of colleagues as primus inter pares. They work much like “two-way windows” [7]: acting on the behalf of colleagues towards executives and as middle managers representing executives towards professionals [31]. Clinical directors are usually appointed for a fixed period and then return to their clinical activity when the mandate expires; usually they are part-time managers who maintain their clinical practice. This organizational form, which is a shift from traditional hospital professional bureaucracies to divisionalized organizational forms [32], was first introduced in the United States in the late 1970’s [33]. It was subsequently adopted by healthcare organizations in many Western countries, including Scandinavia and continental Europe [34]. They have attracted the interest of organizational and health services scholars fascinated by the variety of forms of hybrid professionalism (e.g., [4, 10, 35–37]).

Following initial enthusiasm, critics argued that CDs may pose ethical challenges [38] and exacerbate in-house politics [39] because professionals may be apt to favor their specialty over others [30]. For example, CDs can result in divisionalized professional bureaucracies [24] where traditional professionalism and medical autonomy of the operating core is reinforced rather than reduced, since practicing physicians are not exposed to new managerial work practices. The professional values and interests of doctors remain autonomous from those of management when no increased administrative control or loss of autonomy in decision-making is experienced [24]. As a consequence, one needs to determine whether and to what extent hybrid structures like CDs really do succeed in creating hybrid professionalism, changing what people do and how they do it [40].

Moreover, the relationship between doctors and nurses in CDs hinges on two factors: 1) nurses seem to hold a more positive attitude towards medical management than doctors do [29], and the support nurse managers provide clinical directors may increase their familiarity with managerial behaviors and tools [21]; 2) professional rivalry and turf wars can take place, as CDs can become arenas where management positions are key to strengthening one’s own profession [4].

### Medical Management in Italy

In the Italian National Healthcare System (NHS), the heads of specialty units have historically held leadership positions and played a central role in the strategy building of their units. They had wide legal and managerial responsibility for physical assets (beds, operating rooms, etc.) and staff (both clinicians and nurses). With the advent of the managerialization reforms in 1992, all public hospitals were required to create CDs in their organizational chart and integrate diverse medical specialty units. The chiefs of CDs were selected from among the heads of specialty units. They were practicing physicians who were assigned responsibilities over strategic decision making, budgeting, and clinical governance of the CD [30]. In some instances, CDs had their own budgets which were then divided among the units, while more often resources were negotiated between the chief executive officer (CEO) and each unit chief. Clinical directors were also members on the board of clinical directors, acting mostly as an advisory body for the CEO. Initially, CDs gained traction due to isomorphic pressures rather than rational strategy making [41]. Over time, the effective engagement of doctors as clinical directors increased and CDs were enacted. The aim of the CDs was to overcome the clinical and organizational fragmentation that generated inefficiencies typical of the functional structure built around medical specialty units and widely adopted by Italian hospitals. The CDs had to meet various different organizational needs through the integration of diverse specialty units: 1) better clinical governance via the definition of integrated clinical pathways; 2) reduced inefficiencies and exploitation of economies of scale by sharing physical assets, nursing staff, and technologies; 3) restrict the span of control by CEOs, who interact with a few heads of CDs rather than numerous unit chiefs [30].

In the following paragraph we illustrate the study design and methods, present and discuss our findings, and conclude by highlighting implications for health services management and research.

## Methods

The study setting was the Local Health Authority of Bologna, Emilia Romagna, Italy. Bologna’s LHA has a catchment of 860,000 inhabitants of the Bologna metropolitan area and a staff of about 8000; it manages four hospitals organized in cross-hospital CDs. The cross-hospital CDs were firstly introduced in 2005 to improve the governance of the network and the coordination in decision-making and internal reorganization of the clinical units. On average, each of the eight CDs in place at the time of the study comprised 12 medical specialty units, grouped according to disciplinary proximity and each managed by a unit chief. The CDs were managed by a part-time clinical director who continued practicing as physician and head of one of the specialty units.

For this analysis of how professionals view hybrid professionalism, we used the questionnaire created and validated by Braithwaite and Westbrook [22] to investigate hospital staff attitude toward CDs; the tool has been successfully employed by previous studies on medical management [29, 42]. It is structured in six sections (clinician issues, working relationships, coordination and management, decentralization, organizational performance, final questions, and comments). It was translated into Italian and slightly adapted to the Italian NHS context. Back-translation was performed to check for content accuracy. We added a section on the clinical director’s role and his/her hybrid nature. In this way, we incorporated aspects that had emerged in preliminary interviews with hospital executives and from the literature [43]. The final version of the questionnaire was composed of 65 items: 60 had five possible responses on a Likert-like scale from 5, strongly agree (SA), to 1, strongly disagree (SD).^1^ The intermediate positions on the scale were numbers 4, 3, 2. The descriptors are not numerical; they refer to agree (A), uncertain (U), and disagree (D). The five final items were open questions.

The questionnaire was posted on the LHA intranet with a cover letter that explained the purpose of the survey. The respondents were invited to complete the questionnaire and return it via email between November 2011 and January 2012. Responses were anonymous. The sample was the unit chiefs and nurse managers who worked in one of the eight CDs. The study sample was 123 staff members: the return rate was 47.4% for doctors and 31.6% for nurses; 60% were unit chiefs and 40% nurse managers. Respondents came from different CDs, the majority from the larger CDs. The average age of the sample was relatively high, especially for the doctors, because it was composed of professionals in a managerial position. Moreover, the average age of Italian doctors - irrespective of their organizational role - in 2013 was 51.6 years [44]. The chiefs of the CDs were all men. Table 1 presents a brief profile of respondents.

**Table 1:** Questionnaire Respondent Demographics

	*N* = 123	% female	Predominant age group
Doctors	74	24	50–59 years
Nurses	49	80	40–49 years

For all questionnaire items, the average percentage of staff giving one of the five possible responses was calculated. Following Braithwaite and Westbrook [22], three additional percentages were calculated for each item and then averaged for each questionnaire section. These measured the uncertainty, the intensity, and the polarity of the attitudes the respondents expressed. The polarity index (PI) is particularly relevant as it gives a measure of the spread of the group’s attitudes. An item with low polarity will have responses clustered at either the agree or the disagree end of the scale, whereas an item with high polarity will have a percentage of respondents who agree with the item approximately equal to the percentage of those who disagree.^2^ In previous studies [22, 42] the questionnaire was used to study staff attitudes toward CDs irrespective of a respondent’s profession. For the present study, following Braithwaite and Westbrook [29] we analyzed the responses and report the data in a comparison between doctors and nurses. This was done to understand physicians’ attitudes and to explore similarities and differences between the two groups. In the analysis of the qualitative data we identified concepts in the empirical material and grouped them into categories according to the comments given by the respondents. Data interpretation was performed by iteratively triangulating between quali-quantitative data and organizational theory on hybrids in healthcare.

## Results

Table 2 presents a selection of the most relevant items from each section of the questionnaire. The data from the final section, *Final Questions and Comments*, have been omitted in the interest of brevity.

**Table 2:** Questionnaire Responses by Doctors and Nurses (%). SD denotes strongly disagree, D disagree, A agree, SA strongly agree, PI Polarity Index

	Doctors				Nurses			
	SD + D	U	A + SA	PI	SD + D	U	A + SA	PI
***I - Clinician issues***								
*Involving clinicians in management leads to ethical dilemmas*	61	16	23	38	55	20	24	44
*Involving clinicians in management is wasteful of scarce resources*	74	15	11	15	59	27	14	24
*Clinicians are always able to mobilize powerful forces to resist change*	57	15	28	50	10	27	63	16
*The sharing of professional knowledge and experience has been improved with CDs*	26	23	51	50	20	18	61	33
*By becoming involved in CDs, clinicians have compromised their professional independence*	62	23	15	24	82	14	4	5
***II - Working relationships***								
*Relationship between workplace groups have generally improved with the advent of CDs*	34	11	55	61	27	16	57	46
*There is more conflict between managers and clinicians in a CD structure compared to the traditional structure*	46	22	32	71	49	31	20	42
*Working relationships between executives and managers of CDs are effective today*	23	42	35	65	29	35	37	78
*Working relationships between managers of CDs and their clinical staff are effective today*	27	26	47	57	22	47	31	73
*Working relationships between CDs are effective*	55	23	22	39	65	24	10	16
***III – Coordination and management***								
*Budgets were more easily managed under the traditional organizational structure*	64	19	18	28	55	27	18	33
*CD managers can control clinical activity effectively*	35	31	34	96	24	37	39	63
*Executive managers can better manage costs with the advent of CDs*	7	35	58	12	8	33	59	14
***IV - Decentralization***								
*Traditional organizational structure inhibited the devolution of managerial authority to clinicians*	46	39	15	32	39	45	16	42
*In the past five years, my work has been subject to more intensive monitoring and control*	23	14	64	36	8	35	57	14
*Doctors in this hospital have too much of a say in how things are run*	85	12	3	3	16	47	37	44
*On balance, I prefer the traditional organizational structure*	58	20	22	37	67	31	2	3
***V - Organizational performance***								
*Hospital executives are the major beneficiaries of the introduction of CDs*	24	20	55	44	43	31	27	62
*Doctors are the major beneficiaries of the introduction of CDs*	68	20	12	18	55	35	10	19
*Nurses are the major beneficiaries of the introduction of CDs*	45	23	32	73	71	27	2	3
*The hospital is now more patient-focused because of the CD structure*	59	20	20	34	43	18	39	90
***VI - Clinical director’s role***								
*The CD director has a strong mandate from the executives and can therefore exert credible leadership*	22	38	41	53	20	22	57	36
*The fact that the position of CD director is appointed for a fixed period significantly reduces his/her determination/possibility to manage the CD*	72	11	18	25	47	27	27	57
*The CD director gives precedence to the interests of his/her own discipline over the interests of the CD*	47	24	28	60	37	41	22	61
*The CD director should only have responsibility for clinical governance and organizational issues.*	54	23	23	43	53	22	24	46

One of the main findings from analysis of the first section of the questionnaire was that as far as clinician/professional issues are concerned, the doctors did not see hybrid professionalism as useless (74% disagreed) or as a threat to professional values as it may compromise independence (62%) or lead to ethical dilemmas (61%). Also, they acknowledged that the CDs produced positive professional outcomes, such as increased knowledge sharing (51% agreed). Very similar responses were recorded for the nurses, except for their view on a clinician’s capacity to resist change: the doctors mostly disagreed with this statement (57%), whereas the nurses agreed with it (63%).

The respondents felt that working relationships had improved with the advent of the CDs compared to the conventional organizational model, consistent with the objectives of this new professional model. There was an improvement in relationships between working groups (55%), a reduction in conflict between professionals and managers (46%) – albeit with high polarity (71%) that indicates very different attitudes among doctors – and relationships between the hybrid clinical director and his/her staff were deemed effective (47%). Of note is that nurses’ and doctors’ responses were similar to one another. Furthermore, while the new organizational model was associated with better internal working relationships, it did not succeed in achieving cooperation across directorates (55% reported ineffective working relationships).

The third and the fourth section explored whether professionals perceived an increase in the managerial control of their activity. They agreed that their work was subject to greater control (64%), that budgets were now more easily managed (64%), and that cost management was more effective (58%). Interestingly, despite the greater managerial control and collaboration and integration, the views diverged on a clinical director’s capacity to effectively control clinical activity (34% agreed, 31% uncertain, and 35% disagreed; polarity index 96%).

A particularly interesting response by the doctors was that the conventional organizational structure did not inhibit the devolution of managerial authority to clinicians (only 15% agreed). While it is striking how closely the responses by the Italian respondents aligned with those in the original study conducted in Australia [22], it was one of the very few questions in which the responses diverged. As mentioned above, in the conventional organization in Italian hospitals the unit chiefs held managerial responsibilities, especially for asset management (beds, operating rooms, outpatient departments, etc.), patient flows, scheduling of activities, and human resource management (clinicians and nurses).

It is also relevant to compare the responses by doctors and nurses to the item “*Doctors in this hospital have too much of a say in how things are run*”. While the doctors unanimously disagreed (85%), the nurses either agreed or were uncertain (only 16% disagreed). The fourth section concluded with an overall assessment of the new model, which indicated that the doctors clearly preferred the CDs rather than the conventional professional organizational model (58%) and the nurses preferred the CD model more than the doctors (67%).

In the fifth section, several items explored respondents’ perceptions on the main beneficiaries of the new model. Doctors argued strongly that hospital executives benefitted most (55%), some stated that nurses benefitted (32%), while few responded that the doctors benefitted (12%). While the nurses held strongly positive attitudes toward the new model (see last item in section four), they did not see any specific group as a major beneficiary. Responses to the last item in this section highlighted that the doctors, although they had previously indicated an improvement in collaboration and internal relationships, did not see a direct impact of the new model on patient centeredness (SD + D 59%). This is particularly relevant because it is the second area in which our results differ from those of the original study.

Finally, the sixth section investigated views on the hybrid role of the clinical director. Both doctors and nurses stated that a clinical director has a strong mandate and can exert credible leadership (41% of doctors agreed), also despite his/her fixed-term mandate, and that his position as unit chief does not compromise his capacity to be *super partes*. Furthermore, they agreed that he should have a say in the management of human and financial resources, rather than be responsible only for coordination of care.

When we checked whether there were differences between male and female respondents, we noted that the differences were marginal among the nurses, whereas the few female doctors in the sample were more favorable towards CDs than their male colleagues.

Qualitative analysis of the open answers corroborated the quantitative findings. The majority of respondents held positive or very positive attitudes toward the benefits of new model: greater collaboration, knowledge sharing, clinical integration, and capacity to engage professionals in management and strategy. Some respondents indicated greater transparency as one of the main outcomes of the new model, which achieved partial reform of the traditional informal and tacit processes of communication and decision making typical of professional bureaucracies. Below are a few exemplary quotes:

*“Clinical directorates [ … ] stimulate clinical governance, professional training, the development of clinical guidelines: this engaged us … and this did not happen before” (#52, unit chief)*

*“[now we have] greater awareness of the need for inter-professional integration and to bring together the competencies of multiple disciplines” (#123, unit chief)*

*“Clinical Directorates reduced [clinicians’] self-referentiality, favored professional communication and alignment between professionals and organizational priorities” (#62, unit chief)*

Some of the respondents had more skeptical views, arguing that the new model changed little in the way professionals work or that it was too early to make an assessment. Others were openly critical, stating that doctors had actually reduced their involvement in organizational issues as clinical directors – not necessarily the best professionals – acted as a “filter” between top management and doctors. There was a small but not irrelevant number (about 30%) of professionals proved resistant or at least reluctant towards CDs:

*“Clinical Directorates overall reduced the participation of professionals in management, as it is now in the hands of administrators and clinical directors only” (#44, unit chief)*

*“[this organization] should create the conditions that allow a doctor to work as doctor only, this is the reason he was hired and what he is good at” (#72, unit chief)*

*“the organizational power is now based on a self-attributed capacity to represent other people, rather than on professional competence” (#44, unit chief)*

Furthermore, how the clinical director role is filled by individual professionals does matter:

*“it also depends on the competence of clinical directors, who often is a good doctor or surgeon but may not have the preparation, or the skills, or the time, to effectively perform as a director” (#18, unit chief)*

Finally, analysis of responses to the items investigating the hallmarks of a successful CD demonstrated that it is crucial for both clinicians and nurses to be involved in the managerial practices and strategic issues of a hospital. They claimed:

*“involvement of all the professional roles [ … ] in defining strategic objectives, resources, processes and performance management systems” (#25, unit chief)*

The respondents’ comments also focused on the idea that CDs should act as a middle management line and become the “linking pin” between the strategic apex and the operative core through top-down processes, by endorsing clarity in the devolvement of strategic objectives and responsibilities from senior management to clinicians and nurses, and bottom-up by encouraging debate and dialogue on strategic issues and fostering organizational learning.

## Discussion

Correia and Denis [24] found limited or no impact of CDs on the work of professionals, since clinicians reconfigure managerial criteria according to their own interests. In contrast, the doctors in our sample stated that there was an increase in clinical governance, interdisciplinary collaboration, and standardization of clinical work. Hybridization of work practices did take place, and most interestingly the doctors did not see these changes as a threat to professional values. They perceived that their work is now subject to greater control by managers; Correia and Denis [24] found that medical authority was reinforced rather than reduced, since hybrid managers were unable to strengthen managerial tools or control mechanisms and acted according to professional interests and goals. However, despite greater control, most professionals preferred the new model and did not believe that less self-regulation and autonomy compromises professionalism and its ethos. As a consequence, they did not contest it on the basis of rational or normative elements. They held a much more positive view of CDs than the respondents in Braithwaite and Westbrook’s study [22].

What we found was that, if resistance towards the new model exists, it is rooted in issues of power and domain within the professional organization. Our respondents believe that doctors are the major losers in the new model, while nurses and executives are the major beneficiaries. Although the professional dominance of doctors has been reported worldwide, it is especially strong in Italy where unit chiefs have historically enjoyed a high degree of managerial autonomy. Unlike survey respondents in the UK or Australia, they think that the conventional organizational structure does not inhibit the devolution of managerial authority to clinicians, while the new model jeopardizes their power. Accordingly, if a problem with hybrid professionalism exists, it is not grounded on alleged incompatibility between professional and managerial logics or issues regarding autonomy and professional jurisdiction [11, 45] but rather in the distribution of managerial jurisdiction among professions in healthcare organizations.

Also, we discovered a vast overlap between doctors and nurses in their views on CDs. While Braithwaite and Westbrook [29] found that doctors held negative views towards CDs and nurses had slightly less negative views, we noted positive attitudes in both groups. Qualitative analysis showed that nurses appear capable to support doctors in understanding management. This observation is shared by Sartirana et al. [21] and suggests that doctor-nurse interactions can foster hybrid professionalism. The only differences between the two groups concerned issues of power. While the doctors stated that their influence within the organization was limited and that the new model diminished their power, the nurses had contrary opinions, claiming that doctors have powerful control over organizational forces that oppose processes of change. This reveals how political conflicts can take place within professional organizations, as reported by Spehar et al. [4]. The doctors believed they suffered a progressive loss of power, yet the nurses still perceived them as the most forceful “institutional agents” [46]. Furthermore, our analysis revealed highly polarized attitudes among the doctors. While the majority was positive towards hybridization, others were skeptical, as reported elsewhere (e.g. [10, 47]). Previous studies investigated attitudes by means of qualitative methodologies; the measure of polarity of responses to Braithwaite and Westbrook’s [22] questionnaire provides a quantitative indicator of the phenomenon.

Finally, the underlying premise of hybrid professionalism is that by developing medical management and giving groups of clinicians at the intermediate organizational level responsibility for budgets, organizational, and clinical decisions, conventional ways of rendering professional services can be improved. However, restructuring hospitals into CDs is unlikely in itself to create a “new professionalism” [17]. The challenges of specific contexts need to be understood before implementing change processes to address cultural issues and professional values and behaviors. In this context, we believe that our findings indicate a way forward and provide a basis for further study. Assessment of professionals’ perceptions is the first step to address issues, problems, and opportunities on which healthcare executives can act to improve organizational development and change in their organizations. Our findings on hybrid professionalism show that contingency matters, i.e., that the contextual features of a healthcare system need to be considered in order to make sense of diverse patterns of accommodation between medicine and management [23]. Our findings differ from those reported by studies conducted in the UK, the United States, and Scandinavia, and from the results of the study by Correia and Denis [24]. While it is possible that medicine and management can be brought together, it is also clear that existing power arrangements among professions may hamper the development of new forms of hybrid professionalism.

## Conclusions

The study has a number of limitations as the results could have been influenced by features of the organizational context. Nonetheless, in line with previous studies on the topic [47], we believe that our findings can be representative of the Italian healthcare system, at least in the regions where New Public Management reforms have been embraced. It would be desirable to see studies conducted using different quantitative methodologies, which still are relatively scarce in the field, or studies replicating the questionnaire developed by Braithwaite and Westbrook [22] in other healthcare systems. Furthermore, the data presented here were collected some years ago and may not represent the current situation at the hospital. This limitation notwithstanding, the institutional-legal framework has not changed in the last 10 years and CDs form the main organizational building blocks of healthcare organizations in Italy. Also, the LHA we studied was one of the first in the Italian NHS to establish CDs. This pioneer role makes the LHA of Bologna a perfect context to study the responses of professionals to the CDs in the phase of consolidation. It offers findings for other Italian healthcare organizations that are now developing (not only formally) or consolidating their CDs and also for other healthcare organizations throughout the world which are starting to develop the clinical directorates model and/or are facing similar challenges. The debate surrounding hybrid professionalism has progressed in the last 15 years, and new forms of involvement of doctors in management besides CDs have emerged which introduced matrix-like organizational forms, such as [48]: 1) clinical centers that coordinate care delivery for specific health problem and patient flows (e.g., cardiovascular, neuroscience, orthopedics, frail patients, inflammatory diseases), 2) multidisciplinary and multiprofessional teams across CDs for organizing clinical activity. We believe that this questionnaire provides a valid tool to analyze the views of healthcare professionals towards CDs, which are the main institutional means to involve doctors in management.

Below, we discuss implications that can offer food-for-thought for policy makers and practitioners involved in managing healthcare organizations and professionals. First, the mission and mandate of a CD need to be carefully designed. The CD model is usually meant as an intermediate organizational layer in a hospital’s hierarchy. It is increasingly common to find other forms of integration of clinical specialties, however, in which the aim is to re-organize delivery processes, design and implement clinical pathways, share technologies or physical resources. This is consistent with recent trends in hospital organization that are based on a matrix design: clinical centers, multidisciplinary and multiprofessional teams. This may help to mitigate some power issues which become less problematic when money and higher up hierarchical positions are not involved.

Second, thought should be given to the leadership requisites of hybrid managers in charge of CD governance. For instance, forming a team to manage CDs, as it is not a one-man job. In detail, a strong nursing manager on the team is of paramount importance, as he/she can support clinicians in their transition toward hybrid roles. A critical point, however, is that executives and organizational designers need to carefully monitor how power is distributed across professions within the organization. Conflict between doctors and nurses is not new in healthcare, and our findings show that new hybrid organizational models can exacerbate fractures rather than prevent them.

Third, our findings show that “managers”, whoever they are, seem to be the winner. Clearly, there is a specific point here: whoever manages a CD has to develop specific routines and processes to engage clinicians and health staff in conversation, dialogue, decision-making, evaluation of goals and performance. If professionals perceive that the new model has restricted their participation in management and governance, leaving the real say to executives and clinical directors– as shown in a skeptical quote reported above – it will be difficult to engage them. To give a metaphor, clinicians and health staff may be willing to row if involved in steering the boat. In this context, the responses to questionnaire items investigating coordination and management issues shed light but also cast shadows on the functioning of CDs as a “middle” layer of operations management. If on one hand, respondents thought that CDs generate unprecedented opportunities to develop multidisciplinary and multiprofessional work, on the other there was a high percentage that signaled situations of conflict and silo dynamics across CDs. Again, executives will need to invest heavily in generating opportunities for in-depth engagement of professionals by developing coordination mechanisms and managerial tools - symbols, logos, narratives, story-telling – in order to create a sense of belonging to the hospital at large.

Fourth, while many professionals acknowledged that CDs can facilitate new ways of working, based on multidisciplinarity, teamwork, and multiprofessional integration, they were somewhat pessimistic about whether such improvements actually increased patient-centeredness. This response was not unexpected, as many clinicians and health staff may have felt reassured in having resources and processes under control inside their own unit, just like in the traditional organizational model. Therefore, hybrid managers will need to produce evidence of the benefit of CDs for patients and to document the legitimacy of these hybrid roles and systems in the eyes of professionals.

Some of study limitations were related to the sample demographics and will need to be addressed in future research on the topic. First, the average age of the sample was relatively high because it was composed of professionals in managerial positions who arrived there after many years of work within the organization, especially the doctors. In future, this questionnaire can be advantageously administered to junior professionals not yet in a managerial position. Second, it would be interesting to analyze the differences in the responses by sex and age of the respondents, variables available from the current dataset of the study, but also from the perspective of educational background (e.g., medical specialties) and previous managerial training (e.g., executive management programs) of professionals, as suggested by studies on management education and training in clinical hybrid roles and their influence in achieving specific kinds of performance (mainly, service quality and financial efficiency) in healthcare organizations [49].

Moreover, the COVID-19 emergency can be seen as a watershed between past attitudes to managerial practices in healthcare organizations and a new view of involvement in management. During the pandemic, doctors and nurses alike have been called not only to provide effective and efficient care but also to share in crisis management decisions and actions to tackle the emergency. There are multiple areas of managerial engagement [50]: professionals with managerial responsibility (such as CDs directors) have been involved in the work and the decisions in crisis units; professionals within CDs have had to rapidly adapt their decisions and their organizational processes, convert their organization’s physical assets (beds, operating rooms, intensive care units, etc.), and deal with shortages in resources (e.g., PPE, equipment, invasive and non-invasive ventilators, swab tests), as well as introduce digital technologies for handling outpatient visits and telemonitoring patients at home or in managed facilities (e.g., Patient-Centered Medical Home or Patient Hotels). It would be interesting to analyze the changes in perceptions on CDs via a longitudinal study that could yield interesting insights into the impact of the COVID-19 emergency on the views of professionals about their involvement in CDs and in managerial responsibilities. This will be our future area of focus.

## Data Availability

The dataset of this study is available from the corresponding author on reasonable request.

## Abbreviations

CD: Denotes clinical directorates
LHA: Local Health Authority
CEO: Chief executive officer

## Footnotes

1 Question 25, which in the original questionnaire had a binary answer option, was measured on a Likert-like scale.

2 The polarity index is calculated from the responses of respondents who marked 1, 2, 4, or 5 to the item (i.e., they did not check 3, which indicated uncertainty). The percentage of the smaller group of scores (SA + A or SD + D scores) is divided by the percentage of the larger group of scores (either SA + A or SD + D scores). The polarity index can range from 1, which indicates high polarity (equal proportions of the group agree or disagree with the item), to 0, where all respondents either agree or disagree (i.e., there is consensus).
